# Impact of *Bacillus* on Phthalides Accumulation in *Angelica sinensis* (Oliv.) by Stoichiometry and Microbial Diversity Analysis

**DOI:** 10.3389/fmicb.2020.611143

**Published:** 2021-01-08

**Authors:** Wei-Meng Feng, Pei Liu, Hui Yan, Sen Zhang, Er-Xin Shang, Guang Yu, Shu Jiang, Da-Wei Qian, Jun-Wei Ma, Jin-Ao Duan

**Affiliations:** Jiangsu Collaborative Innovation Center of Chinese Medicinal Resources Industrialization, National and Local Collaborative Engineering Center of Chinese Medicinal Resources Industrialization and Formulae Innovative Medicine, Jiangsu Key Laboratory for High Technology Research of TCM Formulae, Nanjing University of Chinese Medicine, Nanjing, China

**Keywords:** *Angelica sinensis*, rhizosphere microorganism, phthalides, bacterial community, *Bacillus*

## Abstract

Plant-microorganism interaction in the rhizosphere is thought to play an important role in the formation of soil fertility, transformation and absorption of nutrients, growth and development of medicinal plants, and accumulation of medicinal ingredients. Yet, the role that they play in the phthalides accumulation of *Angelica sinensis* (Oliv.) Diels remains unclear. In the present study, we report a correlative analysis between rhizosphere microorganisms and phthalides accumulation in *A. sinensis* from Gansu, China where was the major production areas. Meanwhile, *Bacillus* was explored the potential functions in the plant growth and phthalide accumulation. Results revealed that the common bacterial species detected in six samples comprised 1150 OTUs which were involved in 368 genera, and predominant taxa include *Actinobacteria*, *Acidobacteria*, and *Proteobacteria*. The average contents of the six phthalides were 4.0329 mg/g. The correlation analysis indicated that 20 high abundance strains showed positive or negative correlations with phthalides accumulation. *Flavobacterium*, *Nitrospira*, *Gaiella*, *Bradyrhizobium*, *Mycobacterium*, *Bacillus*, *RB41*, *Blastococcus*, *Nocardioides*, and *Solirubrobacter* may be the key strains that affect phthalides accumulation on the genus level. By the plant-bacterial co-culture and fermentation, *Bacillus* which were isolated from rhizosphere soils can promote the plant growth, biomass accumulation and increased the contents of the butylidenephthalide (36∼415%) while the ligustilide (12∼67%) was decreased. Altogether, there is an interaction between rhizosphere microorganisms and phthalides accumulation in *A. sinensis*, *Bacillus* could promote butylidenephthalide accumulation while inhibiting ligustilide accumulation.

## Introduction

The Chinese medicine producing areas are affected by many factors, such as light, soil, and water. Of the soil environmental factors, the rhizosphere as a micro-environment between the root system and soil interface plays an important role in the soil-root system-microbes interact with each other (Butler et al., 2003). The rhizosphere harbors diverse microbes, community composition and change of rhizosphere microorganisms are important indicators used for measuring soil quality, maintaining soil fertility, and affecting the quality of medicinal materials (Zhou et al., 2015; Xu et al., 2018). Some bacteria can conduce to improve the bioavailability of important mineral elements, such as N, P, and K (Meena et al., 2018); some bacteria can increase the ability of plants to resist stress and suppress soil diseases (Nihorimbere et al., 2012; Haney et al., 2015). Furthermore, it has been found that rhizosphere bacteria also affect the synthesis of officinal effective ingredients. In a previous study on *Baphicacanthus cusia* (NeeS) Bremek, *Burkholderia* was reported to play a major role in the synthesis of indigo (Zeng et al., 2018). In a separate study, the combination of *Piriformospora indica* and *Azotobacter chroococcum* increased the artemisinin content in *Artemisia annua* L (Arora et al., 2016). In another study, the rhizobacterial strain, N5.18, was found to significantly increased hypericin and pseudohypericin in *Hypericum perforatum* (Manero et al., 2012). Research on harnessing rhizosphere microorganisms for medicinal ingredients accumulation was meaningful in sustainable development of Chinese medicine resources.

The roots of *A. sinensis* are commonly used as an herbal drug and have a long officinal history in Chinese, Korean, and Japanese medicines (Sowndhararajan et al., 2017). *A. sinensis* is mainly distributed in Gansu, Yunnan and Sichuan Province, China. Due to the excellent quality, superior clinical effects and good market reputation, the Min County, Gansu Province is the traditional top-geoherb region of *A. sinensis* (Tan et al., 2015). In traditional Chinese medicine, it has functions of enriching blood, promoting blood circulation, relieving pain, and stimulating bowel evacuation, which is famous for its treatment of various gynecological diseases, known as “female ginseng” (Chen et al., 2013; Fang et al., 2014). Since the 1970s, 165 chemical compounds have been separated or identified in *A. sinensis*. Its chemical composition has also been connected to biological activities and clinical applications (Tan et al., 2015). Phthalides are one type of important active ingredient found in *A. sinensis* oil. Common phthalides, including ligustilide, butylphthalide, butylidenephthalide, senkyunolide H, senkyunolide I, and senkyunolide A, are usually used as biomarkers for quality evaluation (Wei and Huang, 2015).

Currently, research on the phthalides of *A. sinensis* mainly focuses on chemical constituent isolation and identification (Lv et al., 2018), quality control (Wei and Huang, 2015), pharmacological activity (Chen et al., 2017), catalytic reaction (Kattela et al., 2018), and practical application (Farias et al., 2018). Meanwhile, rhizosphere microorganism studies of *A. sinensis* mainly focus on investigating community changes and the effect of continuous cropping soil (Jiang et al., 2009; Zhang et al., 2015). However, our understanding and systematic research on the correlation between rhizosphere microorganisms and phthalides accumulation remain insufficient.

In this study, the main purpose was to reveal the correlation between rhizosphere microorganisms and phthalides accumulation from Gansu province. The ultra-high performance liquid chromatography-tandem mass spectrometry (UHPLC-MS/MS) method was utilized to determine the content of six phthalides in *A. sinensis* which were collected from Gansu. Meanwhile, Illumina MiSeq high-throughput sequencing technology was used to investigate the diversity and composition of rhizosphere microorganisms. A relationship analysis was conducted in order to reveal a correlation between rhizosphere microorganisms and phthalides accumulation. Then, the evaluation of the effect on the phthalides accumulation of two strains of *Bacillus* isolated from rhizosphere soils by tissue culture seedling co-culture and fermentation experiment. Thus, it could provide clues to further explore the influence of microorganisms on the medicinal materials quality.

## Materials and Methods

### Plant Materials and Reagents

The fresh roots of biennial *A. sinensis* were collected from Min Country, Dingxi city, Gansu Province, China (E 103.8571, N 34.5190, H 2943 m). The roots were authenticated by the corresponding author, Dr. Hui Yan. Roots were packed in sterile plastic bags and stored at −20°C for further analyses. Soil samples were collected from six random locations within the same origin. All collected soil samples were cleared of plant roots, leaf debris, and others, then stored at −80°C after flash freezing with liquid nitrogen for further analyses (Table 1).

**TABLE 1:** *A. sinensis* samples information.

**Sample**	**Sample no.**	**Location**	**Longitude/latitude**	**Altitude/m**
Roots/ Rhizosphere soils	M-ZC2-G-1 M-ZC2-G-2 M-ZC2-G-3 M-ZC2-G-4 M-ZC2-G-5 M-ZC2-G-6	Min Country, Dingxi city, Gansu Province	103.8571/ 34.5190	2943

### Sample Preparation

Fresh samples were cut into small pieces (particle size < 0.5 cm), and 0.5 g fresh root was accurately weighed. Then, 20 mL methanol was added to a 50 mL conical flask with sonication at 100 Hz and 25°C for 45 min. After extraction when the samples were weighed, methanol was added if any weight loss was observed (Zhu et al., 2017). The supernatant on the sample plate was obtained by centrifugation at 13,000 r/min for 10 min and filtered through a 0.22 μm membrane filter then stored at 4°C before ultra-high performance liquid chromatography (UHPLC) analyses were conducted. The standard solution was prepared by mixing the six standard stock solutions, except clarithromycin, in equal amounts. Then, the solution was diluted with methanol to a suitable concentration for determining and constructing a standard curve. A detailed description of the six standard stock solutions was available in the Supplemental Experimental Procedures.

### UHPLC-QqQ-MS Analysis

Chromatographic analyses were performed using a Waters Acquity UPLC system (Waters, Corp., MA, United States). Mass spectrometry (MS) was carried out using an AB SCIEX Triple Quad 6500 plus (AB SCIEX Corp., MA, United States) with electrospray ionization (ESI). The methodology validation and detailed description of chromatographic condition were showed in the Supplemental Experimental Procedures. All samples that removed moisture interference were prepared according to section 2.2; identification of the target peaks was performed by comparing their UHPLC retention times and mass/charge ratios (*m/z*) with the standards (Zhang et al., 2019). The concentration of each sample was calculated by an internal standard comparison method.

### PCR Amplification and Illumina MiSeq Sequencing

Microbial DNA was extracted from soil samples by using an E.Z.N.A.^®^ soil DNA Kit (Omega Bio-tek, GA, United States) following the manufacturer’s instructions. DNA concentration and purification were determined by using a NanoDrop 2000 UV-vis spectrophotometer (Thermo Scientific, DE, United States). DNA quality was checked by 1% agarose gel electrophoresis. An Illumina Miseq platform (Illumina, CA, United States) was used to measure the diversity and composition of the bacterial community. An equimolar amount of the purified PCR products were submitted to amplicon library on an Illumina MiSeq platform (Illumina, CA, United States) following the Shanghai Majorbio Bio-Pharm Technology Co. Ltd. (China) standard protocols (Yang and Wang, 2018). Sequencing was performed using Illumina Miseq PE300 platform (Shanghai Majorbio Bio-Pharm Technology Co., Ltd.). The datasets generated during the current study were deposited and are available at the National Center for Biotechnology Information (NCBI), Sequence Read Archive (SRA), under the accession number PRJNA555671^1^.

### Amplicon Data Analysis

Raw fastq files were demultiplexed and quality-filtered by Trimmomatic, then merged by Flash v1.2.11^2^. Operational taxonomic units (OTUs) were clustered with 97% similarity cutoff using UPARSE v7.0^3^. Chimeric sequences were identified and removed using UCHIME. The taxonomy of each 16S rDNA sequence was analyzed using the RDP Classifier v2.2^4^ algorithm against the Silva 16S rDNA database (Release128^5^) using a confidence threshold of 70% (Qu et al., 2018; Zhang et al., 2018). These sequences were clustered to OTUs with 97% sequence identity using Mothur software. OTUs were used for alpha diversity and beta diversity by QIIME v1.9.1^6^. Alpha-diversity analysis is mainly for intra-sample comparison, including species dilution curve, richness and diversity index (Ace, Chao1, Simpson, Shannon index), etc., calculated by the Mothur component based on the OTUs level. Beta-diversity analysis is mainly a comparison between samples or groups. Correspondence diagrams between samples and bacteria are made by Circos^7^ which reflects the proportion of dominant bacteria in each sample. The PCoA analysis steps are as follows: first calculate the diversity matrix in the QIIME software according to the sample OTUs file and the evolutionary tree file, and the distance between the samples uses Bray-Curtis and weighted-unifrac, respectively, distance algorithm; then according to the distance matrix in the R software (V3.2.3) output graphics. All data analyses were conducted using the cloud platform^8^ provided by Shanghai Majorbio Bio-Pharm Technology Co., Ltd.

### Co-culture of Different Bacterial Solutions With Tissue Culture Seedlings of *A. sinensis*

Two stains bacterial, XG-2 and XG-3, isolated from the *A. sinensis* soils of Min Country were identified as the *Bacillus subtilis* and *Bacillus velezensis*, based on 16S rDNA sequence. Seeds of *A. sinensis* which were collected from Min Country, Dingxi city, Gansu Province, China, were used in experiments. 20 μL sterile distilled water, 20 μL of XG-2-JY/XG-3-JY and XG-2-J/XG-3-J was added to each plant rhizosphere and cultivation was continued in the same condition when 30 to 40 days which the cotyledons were flattened and true leaf had grown were after planting. Each treatment was performed on 12 plants. In the colonization experiment, *A. sinensis* were collected at 7 days. Two plants as one sample was replicated in the experiment. Plant height were measured by using a precision straight edge (Deli, China). A whole plant was determined the fresh weight. Reference the method of “UHPLC-QqQ-MS Analysis” determined the phthalides contents. The culture conditions of bacterial and tissue culture seedling were available in the Supplemental Experimental Procedures.

### Fermentation

Powders of *A. sinensis* were the organic nitrogen and carbohydrate source in the fermentation medium. The solid-state fermentation (XG-2-G/XG-3-G) was composed of 4 g *A. sinensis* powders, 1 g wheat bran, 6 mL sterile distilled water, and 1 mL bacterium liquid culture (Xia et al., 2017). The liquid fermentation (XG-2-Y/XG-3-Y) was composed of 5 g *A. sinensis* powders, 95 mL sterile distilled water, and 5 mL bacterium liquid culture. 1/5 mL sterile distilled water were added into fermentation medium as control-G/control-Y. All mixtures except bacterium liquid culture were sterilized at 121°C for 15 min. The cooled samples were incubated at 30°C with constant shaking at 160 rpm for 7 days after inoculating bacterium liquid culture. We set up control group with sterile distilled water. The mediums were dried to constant weight before content analysis. All experiments were divided six groups and three replications per treatment. Reference the method of “UHPLC-QqQ-MS Analysis” determined the phthalides contents.

### Data Processing and Statistical Analyses

Student’s *t*-test of the phthalides content analysis and growth parameters was performed with SPSS v21.0. Differences between groups were considered significant when *p* < 0.05. The top 50 abundant genera and samples and the Spearman correlation coefficients of the top 50 abundant genera and phthalides accumulation were calculated and displayed on a heat map. Spearman correlation coefficients were calculated using R v3.5.1. The correlation coefficient is always between −1 and 1; the closer the correlation to ±1, the closer it is to a perfect linear relationship (Liu et al., 2017). Results was built and optimized using GraphPad Prism 7.0, which were presented as the mean ± standard deviation (SD).

## Results

### Diversity of the Soil Bacterial Community

**FIGURE 1:**
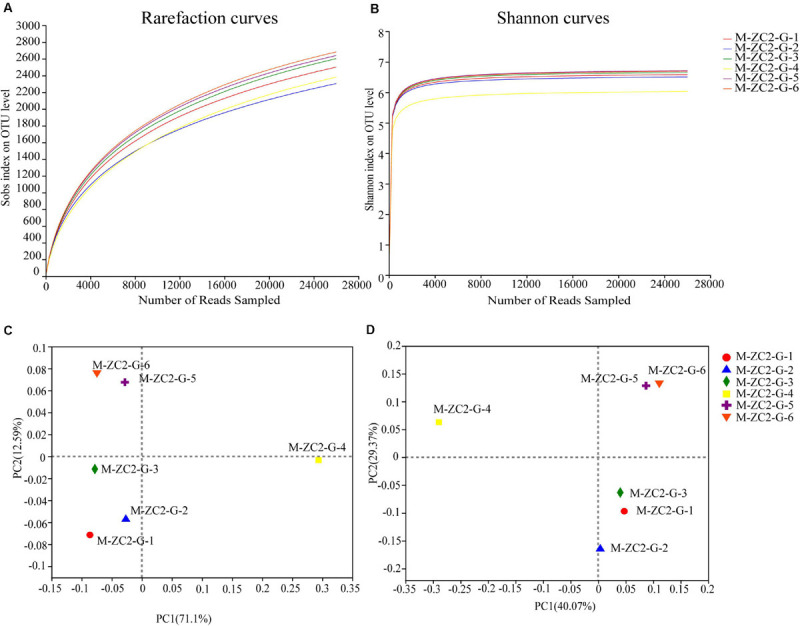
Alpha and Beta diversity analyses of sample soils. **(A)** Rarefaction curve from sobs index, **(B)** Shannon-Wiener curve, **(C)** PCoA analysis based on Bray-Curtis, and **(D)** PCoA analysis based on weighted-unifrac.

The alpha bacterial community diversity indices, such as Sobs, Shannon, Simpson, Chao, Ace and Coverage were provided (Table 2). The Sobs means the observed richness in the samples. The Chao and Ace were the indexes to estimate the number of OTUs contained in the sample. The Shannon and Simpson were the indexes to estimate the community diversity in the sample. The larger Shannon index means higher community diversity, but the larger Simpson index means the lower community diversity. The M-ZC2-G-4 samples exhibited lower community diversity compared to other samples. The rarefaction curve (sobs index) showed the richness of species and plausibility of sequencing data in samples; as the rarefaction curves tended to approach saturation, it indicated the rationality of the data (Figure 1A). The Shannon-Wiener curve tended to be flat, and the amount of sequencing data was large enough to reflect the vast majority of microbial information in the samples (Figure 1B). Good’s coverage estimates for all soil samples were >0.95 and represented good sampling depth.

**TABLE 2:** Diversity indices of the soil bacterial community in *A. sinensis* samples.

Samples*	**Sobs**	**Shannon**	**Simpson**	**Ace**	**Chao**	**Coverage**
M-ZC2-G-1	2501	6.5828	0.0039	3262.0790	3333.3562	0.9700
M-ZC2-G-2	2304	6.5043	0.0040	3061.1232	3088.4386	0.9718
M-ZC2-G-3	2603	6.6529	0.0037	3415.2336	3434.1108	0.9684
M-ZC2-G-4	2381	6.0286	0.0248	3221.0587	3137.9617	0.9694
M-ZC2-G-5	2640	6.7091	0.0034	3386.6116	3411.7908	0.9694
M-ZC2-G-6	2683	6.6928	0.0042	3447.2851	3420.6199	0.9689

*Sobs, the observed richness; Shannon, the Shannon diversity index; Simpson, the Simpson diversity index; Ace, the ACE estimator; Chao, the Chao1 estimator; Coverage, the Good’s coverage.

In order to analyze the differences in six soils, a principal coordinate analysis (PCoA) was conducted (Zhou et al., 2017, 2018). All data were normalized using SPSS. The PCoA analyses were based on Bray-Curtis and weighted-unifrac (Figures 1C,D). Similarity distances revealed that M-ZC2-G-4 was widely separated from the other Gansu samples (Bray-Curtis PC1 = 71.1%, PC2 = 12.59%; weighed-unifrac PC1 = 40.07%, and PC2 = 29.37%). The alpha bacterial community diversity indices were showed that the M-ZC2-G-4 sample exhibited lower community diversity compared to other samples. This also has an expression in beta bacterial community diversity which M-ZC2-G-4 was widely separated from the other Gansu samples.

### Soil Bacterial Community Composition

A total of 302613 high-quality 16S rDNA sequences were obtained from 6 soil samples. These sequences were distributed among 4107 different OTUs at 97% similarity (Supplementary Table 1). Bacterial species detected in these samples comprised 36 phyla, 87 classes, 168 orders, 333 families, 648 genera, and 1431 species.

**FIGURE 2:**
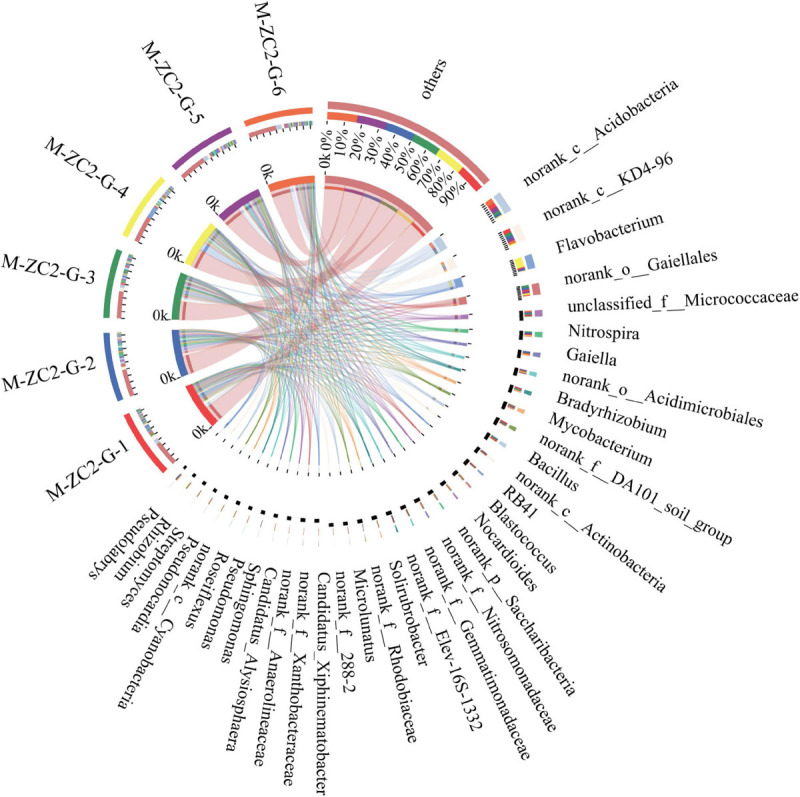
Core bacterial of the *A*. *sinensis* rhizosphere microbiota.

The bacterial composition was analyzed at the phylum and class level (Supplementary Figure 1). Results revealed that the soils were dominated by *Actinobacteria*, *Proteobacteria, Chloroflexi*, *Acidobacteria*, and *Bacteroidetes*. On the class level, the dominant strains were *Actinobacteria*, *Alphaproteobacteria, Acidobacteria*, *Betaproteobacteria*, and *KD4-96*. The Venn diagram revealed that 1150 OTUs of 368 genera were common in Gansu soils (Supplementary Figure 2). Results indicated that the diversity of bacterial composition was similar in the six rhizosphere soils. The common bacteria mainly concentrated on 27 phyla, *Actinobacteria*, *Proteobacteria, Chloroflexi*, *Acidobacteria* and *Bacteroidetes*, etc. *Gracilibacteria* has only existed in the M-ZC2-G-6 sample. No specific strain exists in other samples on phylum level. The relative abundance of bacteria in the rhizosphere soil was then analyzed on the genus level; the top 50 relatively abundant bacteria are mainly presented (Supplementary Figure 3). Analyze the dominant bacteria in each sample by Circos (Figure 2), the core top 10 bacteria with a relatively high abundance were *Flavobacterium*, *Nitrospira*, *Gaiella*, *Bradyrhizobium*, *Mycobacterium*, *Bacillus*, *RB41*, *Blastococcus*, *Nocardioides*, and *Solirubrobacter*.

### Quantification of the Phthalides From Gansu Samples

**FIGURE 3:**
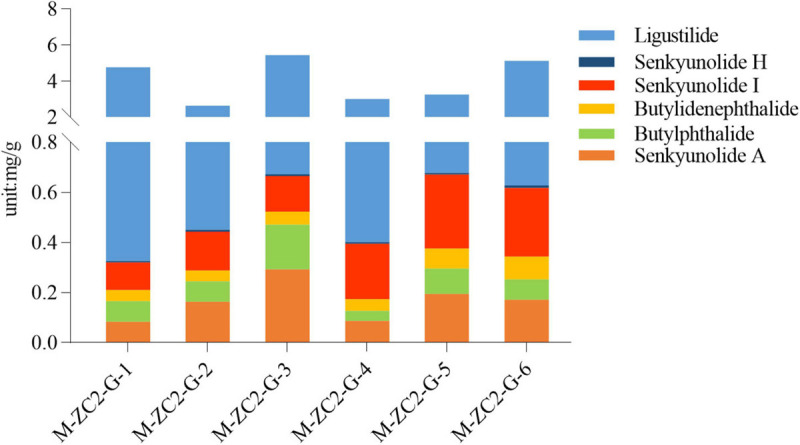
Content levels of the tested components across samples.

Ultra-high performance liquid chromatography-QqQ-MS was applied to simultaneously determine six markers in 12 samples of *A. sinensis* (Figure 3 and Supplementary Table 2). The most selective and specific transition was chosen for MRM determination, and all MRM parameters are provided (Supplementary Table 3). The UHPLC method was validated by assessing linearity, precision, stability, LOD, LOQ, and recovery (Supplementary Table 4).

The content of ligustilide was highest among the six components in all samples (2.1857–4.4892 mg/g). The contents of the six phthalides were 4.0329 mg/g. The average contents of ligustilide, butylphthalide, butylidenephthalide, senkyunolide H, senkyunolide I and senkyunolide A were 3.5130, 0.0940, 0.0593, 0.0064, 0.1996, and 0.1607 mg/g, respectively.

### Correlation Between Rhizosphere Microorganisms and Phthalides Accumulation

**FIGURE 4:**
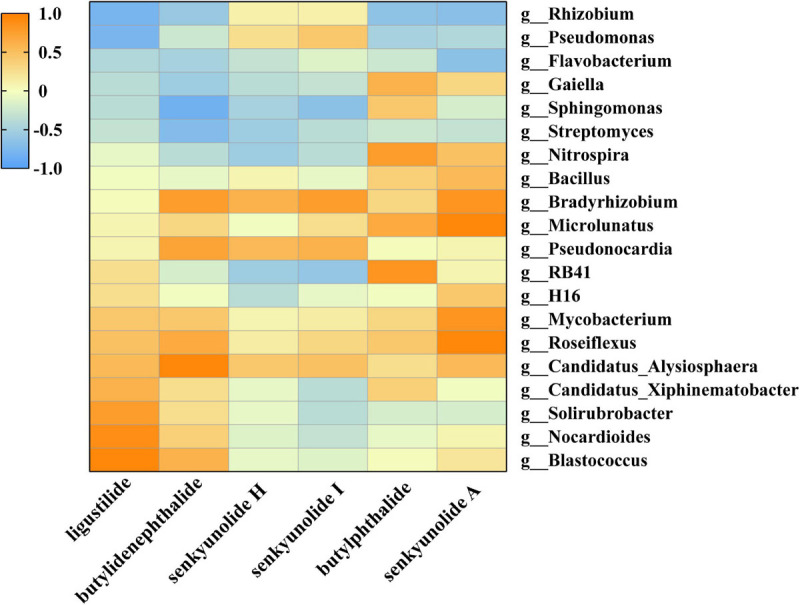
The 20 strains that exhibited a significant positive/negative correlation with phthalides accumulation.

The Spearman correlation coefficient of phthalides accumulation and the top 50 abundant bacteria on genus level were calculated. Results revealed that 20 strains (Figure 4) which were concentrated in *Proteobacteria*, *Actinobacteria* and *Chloroflexi* exhibited the positive/negative correlations with phthalides accumulation.

*Candidatus_Alysiosphaera, Roseiflexus, Mycobacterium, Pseudonocardia*, and *Bradyrhizobium* exhibited a positive correlation with six phthalides. On the contrary, *Streptomyces* and *Flavobacterium* exhibited a negative correlation with six phthalides. Of course, some strains show different effects on the six phthalides. Several bacteria, including *Rhizobium* and *Pseudomonas* exhibited a negative correlation with ligustilide, butylidenephthalide, senkyunolide A and butylphthalide while they were showed a positive correlation with senkyunolide H, senkyunolide I. Some strains, such as *Nitrospira* and *Bacillus* exhibited a negative correlation with ligustilide, butylidenephthalide and senkyunolide I, but they also showed a positive correlation with senkyunolide A and butylphthalide. These results suggest that the rhizosphere bacteria were involved in six phthalides accumulation (Supplementary Table 5), which indicates that these can be used as important biomarkers when the correlation between the rhizosphere microorganisms and phthalides accumulation is considered.

### Plant Growth Parameters and Phthalides Contents

**FIGURE 5:**
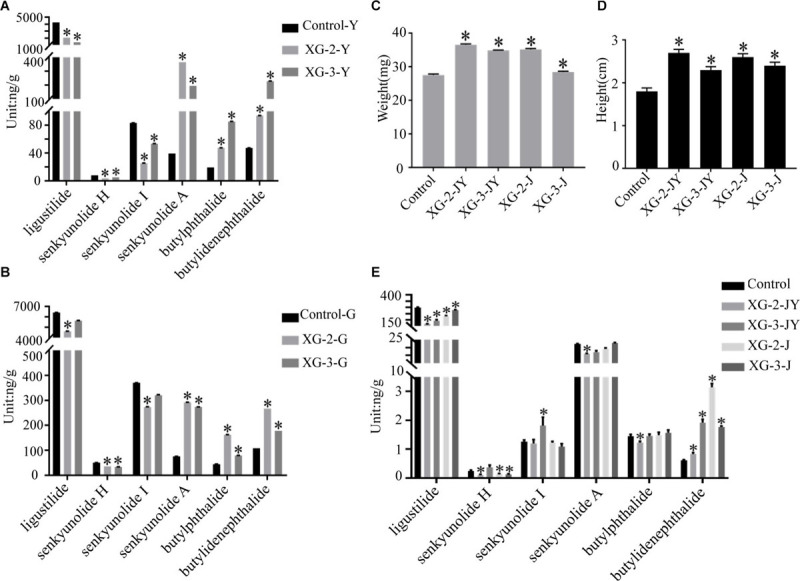
The plant growth parameters and phthalides contents. **(A)** Phthalides contents of liquid fermentation, **(B)** phthalides contents of solid-state fermentation, **(C)** plant heights of tissue culture seedlings, **(D)** plant fresh weights of tissue culture seedlings, **(E)** phthalides contents of issue culture seedlings. Asterisks indicate significant differences relative to control (^∗^*p* < 0.05, Student’s *t*-test).

*Bacillus subtilis* (XG-2) and *Bacillus velezensis* (XG-3) bacterial cell suspension addition all significantly increased the plant heights and fresh weights (Figure 5). The average plant heights and fresh weights in the co-culture with XG-2-JY (2.73 cm, 36.56 mg)/XG-3-JY (2.28 cm, 34.87 mg) and XG-2-J (2.57 cm, 35.17 mg)/XG-3-J (2.40 cm, 28.46 mg) was significantly higher than the control samples (1.75 cm, 27.55 mg) (*p* < 0.05). The result proved that *Bacillus* strains had a function in growth promoting of *A. sinensis.*

The effect of phthalides accumulation by co-culture and fermentation showed *Bacillus subtilis* and *Bacillus velezensis* could increase the contents of the butylidenephthalide while the ligustilide was decreased. The average content of ligustilide in the XG-2-JY/J/Y/G (169.9205, 230.7876, 2083.6412, 4634.7486 μg/g, respectively) and XG-3-JY/J/Y/G (193.8898, 44.9352, 1400.9418, 5661.0393 μg/g, respectively) groups was significantly lower than the control/Y/G (298.52, 4268.0747, 6420.3539 μg/g) (*p* < 0.05 except XG-3-G). The average content of butylidenephthalide in the XG-2-JY/J/Y/G (0.8290, 3.1409, 94.0077, 267.2646 μg/g, respectively) and XG-3-JY/J/Y/G (1.9195, 0.8703, 229.3146, 178.1602 μg/g, respectively) groups was significantly higher than the control/Y/G (0.6087, 47.2929, 108.3795 μg/g) (*p* < 0.05). The results revealed that *Bacillus subtilis* and *Bacillus velezensis* play an important role in plant growth and different phthalides accumulation of *A. sinensis.*

## Discussion

For the soil bacteria community and diversity of rhizosphere microorganisms from Gansu soils in this study, core bacterial of the *A. sinensis* rhizosphere microbiota by Circos analysis were *Flavobacterium*, *Nitrospira*, *Gaiella*, *Bradyrhizobium*, *Mycobacterium*, *Bacillus*, *RB41*, *Blastococcus*, *Nocardioides*, and *Solirubrobacter*. *Actinobacteria* which were a group of important plant-associated spore-forming bacteria were well-known as dominant phyla in soil (Novello et al., 2017). In our result, half of the core bacterial, including *Gaiella*, *Mycobacterium*, *Blastococcus*, *Nocardioides*, and *Solirubrobacter*, belong to *Actinobacteria*. Many researches have reported for their biocontrol, plant growth promotion, and interaction with plant (Palaniyandi et al., 2013). The study of the biosynthesis of phthalides, which began with the structural determination of mycophenolic acid, was showed the phthalide fragment derived from the polyketide pathway (Leon et al., 2017). Specially, *Mycobacterium*, and *Nocardioides* which show a positive correlation with phthalides accumulation were found to be with the potential for producing polyketides (Saxena et al., 2003; Sun et al., 2012). Moreover, *Nitrospira* play an important role in Nitrification which is a central nitrogen (N) cycling process, the sequential aerobic oxidation of ammonia to nitrate via nitrite (Koch et al., 2019). *Bacillus* are known to contain plant growth promoting bacteria with being able to produce ammonia, siderophores, catalase, indole acetic acid (Khan et al., 2019) and antibiotics against soilborne pathogenic fungi (Cazorla et al., 2007). *Flavobacterium* had the function of degradation of organophosphorus compounds (Singh and Walker, 2006). The various functions of the strains may be the reason why they become core bacteria in *A. sinensis* soils.

In this study, two strains, *Bacillus subtilis* and *Bacillus velezensis*, were isolated from the rhizosphere soil of *A. sinensis. Bacillus* was one of the core top 10 abundant strains on genus level which was exhibited correlations with phthalides accumulation. In the treatment experiments, the results indicated *Bacillus* has a significant positive effect on the plant heights and fresh weights. The previous study has reported that *Bacillus* were found to possess nitrogen fixing, phosphorous solubility and IAA biosynthesis activities to promote the growth of black pepper (Lau et al., 2020). These characteristics of *Bacillus* were its possible way of promoting the growth of *A. sinensis* tissue culture seedling. Hence, *Bacillus* can act as an alternative to plant growth enhancement agrochemicals in the field cultivation of *A. sinensis*. However, as a Chinese medicinal material, it was also necessary to consider the accumulation of its medicinal active ingredients during the growth process. In present study, *Bacillus subtilis* and *Bacillus velezensis* could increase the contents of the butylidenephthalide while the ligustilide was decreased. Although the result was consistent with the correlation analysis, it had some certain questions in practical applications. For the influence of phthalides accumulation suggested that we should pay attention to the addition quantity when using *Bacillus* as a bio-fertilizer of *A. sinensis*.

Many researches proved the plant growth promoting rhizobacteria (PGPR) as a biological fertilizer could stimulate plant growth and component accumulation (Nawaz et al., 2020). Based on the correlation analysis and results of co-culture and fermentation, the interaction between rhizosphere microorganisms and phthalides accumulation may depend on two possible pathways, direct and indirect mechanisms. In terms of direct mechanisms, bacteria colonize the plant and produced the components, such as some enzymes, that were involved in the synthesis of ligustilide and other components. In terms of indirect mechanisms, bacteria produced some components in rhizosphere, but only when the components were absorbed and utilized by plants, it could participate in phthalide accumulation. For example, the mechanism of biological control reduced the impact of diseases by the rhizobacteria production of siderophores and protection enzymes, such as chitinase, glucanase, and ACC-deaminase (Ongena and Jacques, 2008; Vejan et al., 2016). Notably, a plant’s own secretions will affect rhizosphere microorganisms. A previous report was showed that ligustilide at a 100 μg/mL concentration was confirmed against *Bacillus* (Chou et al., 2006). Moreover, *Bacillus* exhibited high degradation efficiency of a polycyclic aromatic hydrocarbons (PAHs) mixture of naphthalene, fluorene, phenanthrene, anthracene, and fluoranthene in soils heavily contaminated with crude oil (Ma et al., 2018). It may be the high degradation and moderate inhibition ability exhibited by *Bacillus* that contributes to its negative correlation with ligustilide. Due to the lack of research on phthalides biosynthetic pathways, the mechanism of *Bacillus* effect on phthalides accumulation was still unclear which should need a further exploration.

## Conclusion

Overall, the rhizosphere microorganisms and phthalides accumulation indicated that rhizosphere microorganisms may play an important role in phthalides accumulation, meanwhile, phthalides also was showed an effect on bacteria growth. These findings will serve as supplemental information regarding the rhizosphere microorganisms of *A. sinensis* and are of great scientific significance with a potential applicable value in the rhizosphere of *A. sinensis*. Although the result provided some insight of the rhizosphere microorganisms as the bio-fertilizer in order to the sustainable development of *A. sinensis*, the interaction between rhizosphere microorganisms and phthalides accumulation will only be understood when we have better comprehension in the potential functions of rhizosphere microorganisms and the information of phthalides biosynthesis. In the long run, this information could assist the screening of beneficial probiotics in the rhizosphere as the bio-fertilizer in order to improve the yield and phthalides accumulation and promote sustainable development of the *A. sinensis*.

## Data Availability Statement

The datasets presented in this study can be found in online repositories. The names of the repository/repositories and accession number(s) can be found below: https://www.ncbi.nlm.nih.gov/, PRJNA555671.

## Author Contributions

W-MF, PL, HY, and J-AD conceived and designed the experiments. W-MF and J-WM performed the experiments. W-MF, PL, E-XS, and D-WQ analyzed the data. J-AD, D-WQ, SZ, GY, and SJ contributed reagents, materials, and analysis tools. W-MF and PL wrote the manuscript. All authors have read and approved the final manuscript.

## Conflict of Interest

The authors declare that the research was conducted in the absence of any commercial or financial relationships that could be construed as a potential conflict of interest.

## Footnotes

1 https://www.ncbi.nlm.nih.gov/bioproject/PRJNA555671

2 https://ccb.jhu.edu/software/FLASH/index.shtml

3 http://drive5.com/uparse/

4 http://sourceforge.net/projects/rdp-classifier/

5 http://www.arb-silva.de

6 http://qiime.org/install/index.html

7 http://circos.ca/

8 https://www.i-sanger.com/
