# Supplementary material for: Impact of Bacillus on Phthalides Accumulation in Angelica sinensis (Oliv.) by Stoichiometry and Microbial Diversity Analysis
Source: Front Microbiol. 2021 Jan 8;11:611143. doi: 10.3389/fmicb.2020.611143 (PMC7819887; doi:10.3389/fmicb.2020.611143)
Supplement: Supplementary file 1 [file Data_Sheet_1.docx]

**Figure S1** The community abundance on phylum and class level of samples


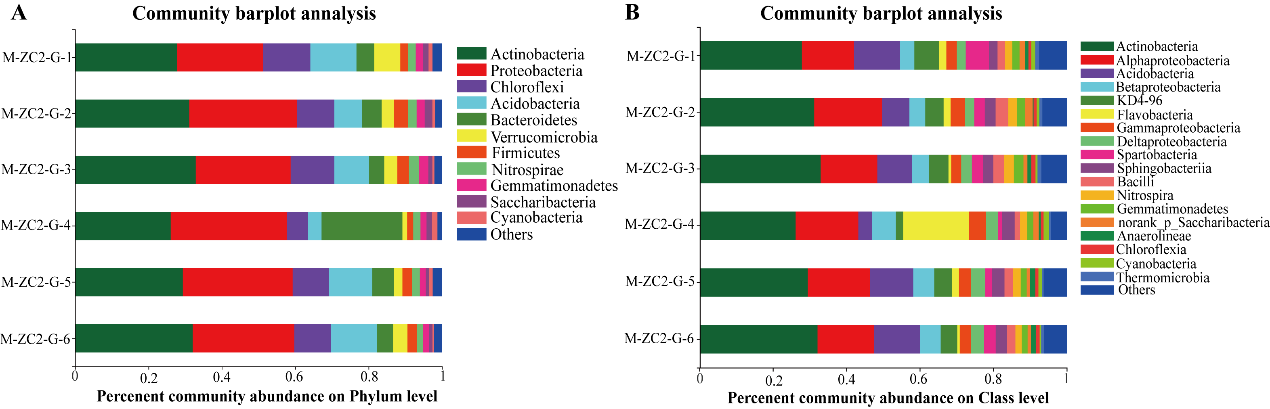


**Figure S2** The Venn map from soils on genus and OUT level


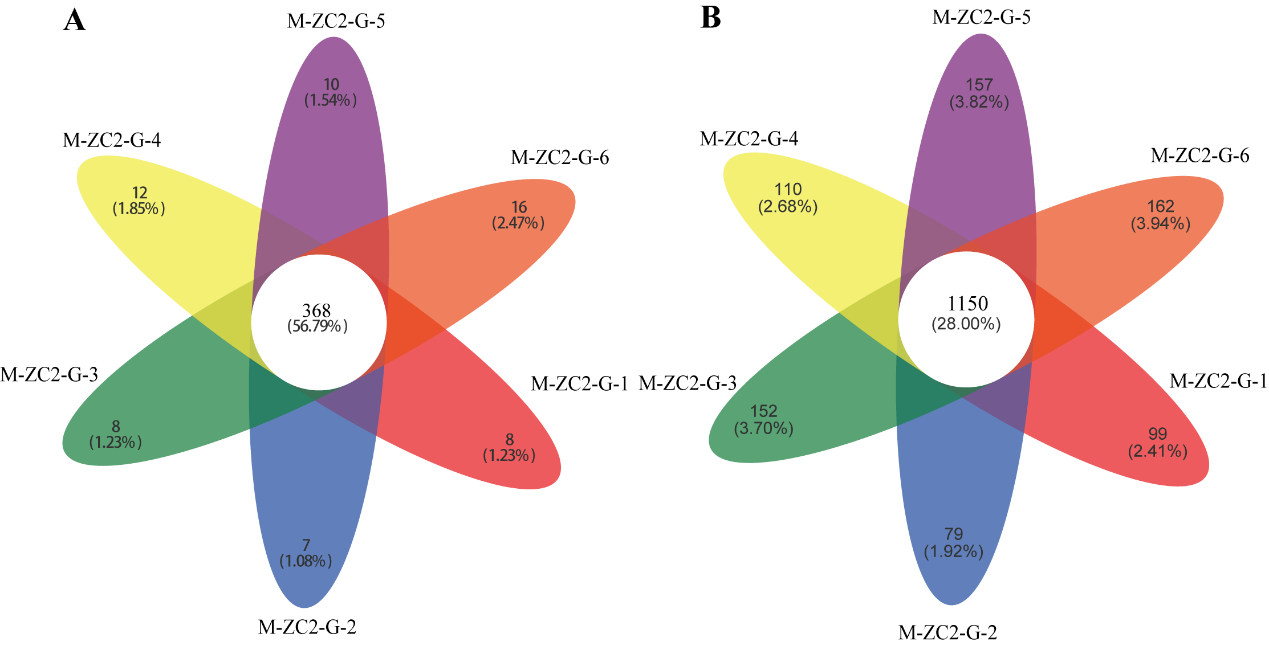


**Figure S3** The top 50 relative abundance of samples on genus level


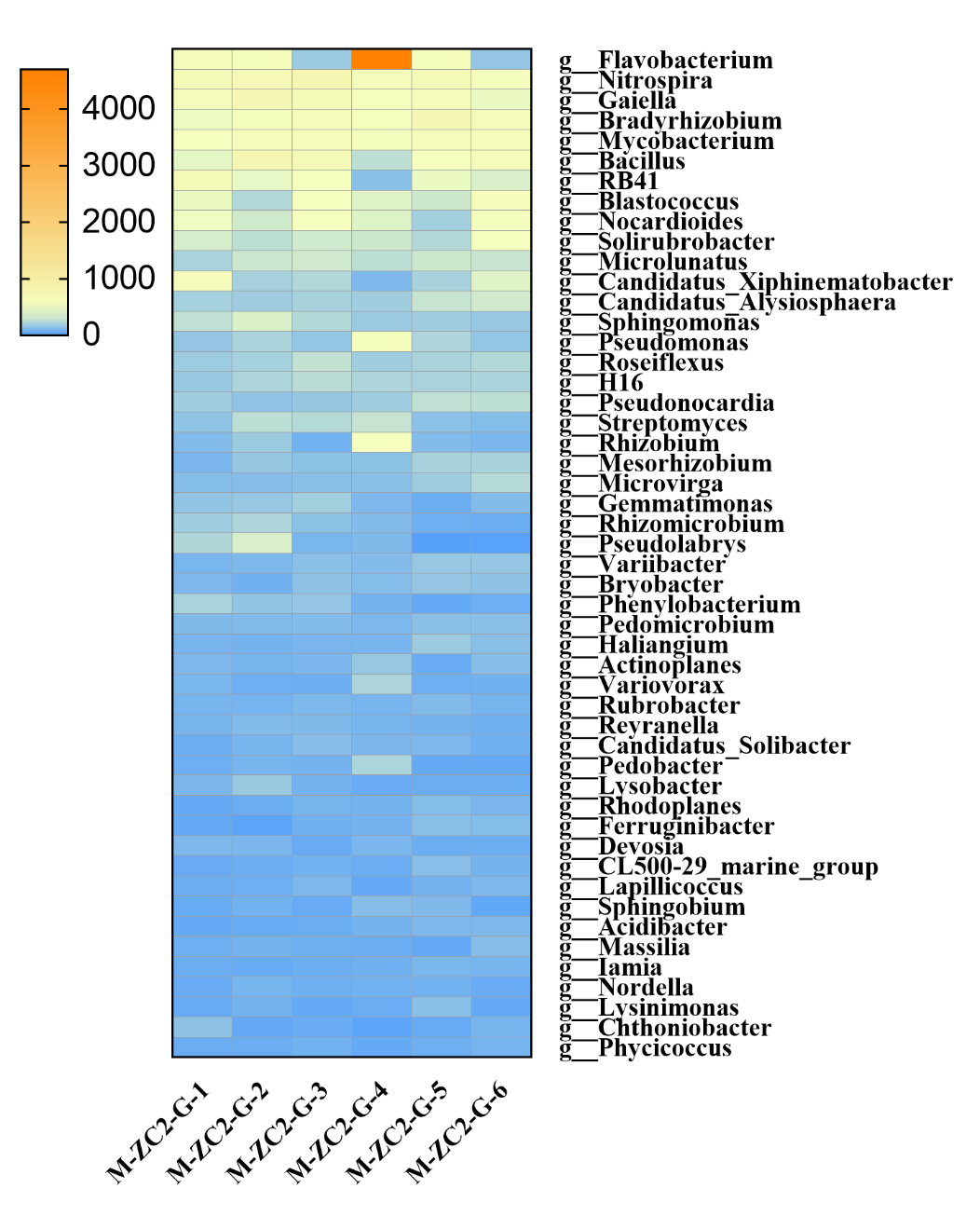


**Table S1** Summary for *Illumina* *MiSeq sequencing* information of samples

| SampleInfo | Seq_num | Base_num | Mean_length | Min_length | Max_length |
| --- | --- | --- | --- | --- | --- |
| M-ZC2-G-1 | 53599 | 23475919 | 437.9917 | 331 | 482 |
| M-ZC2-G-2 | 49735 | 21754492 | 437.4081 | 324 | 481 |
| M-ZC2-G-3 | 44900 | 19649094 | 437.6190 | 269 | 496 |
| M-ZC2-G-4 | 56341 | 24694651 | 438.3069 | 298 | 480 |
| M-ZC2-G-5 | 45060 | 19726110 | 437.7743 | 279 | 512 |
| M-ZC2-G-6 | 52978 | 23192310 | 437.7725 | 318 | 494 |

**Table S2** Content (mg/g) levels of the tested components across samples. (n=6)

| samples | ligustilide | butylphthalide | butylidenephthalide | senkyunolide H | senkyunolide I | senyunolide A | sum |
| --- | --- | --- | --- | --- | --- | --- | --- |
| M-ZC2-G-1 | 4.4389 | 0.0826 | 0.0445 | 0.0048 | 0.1097 | 0.0837 | 4.7642 |
| M-ZC2-G-2 | 2.1857 | 0.0811 | 0.0431 | 0.0063 | 0.1553 | 0.1496 | 2.6209 |
| M-ZC2-G-3 | 4.7722 | 0.1793 | 0.0518 | 0.0046 | 0.1422 | 0.2917 | 5.4419 |
| M-ZC2-G-4 | 2.6116 | 0.0392 | 0.0470 | 0.0054 | 0.2203 | 0.0874 | 3.0108 |
| M-ZC2-G-5 | 2.5801 | 0.1013 | 0.0788 | 0.0075 | 0.2950 | 0.1950 | 3.2578 |
| M-ZC2-G-6 | 4.4892 | 0.0804 | 0.0906 | 0.0098 | 0.2749 | 0.1569 | 5.1018 |

**Table S3** The MRM transitions and parameters applied of six components

| Name | Formula | RT min | m/z precursor ion | m/z product ion | DP | EP | CE | CXP |
| --- | --- | --- | --- | --- | --- | --- | --- | --- |
| senkyunolide A* | C12H16O2 | 7.51 | 193.027 | 136.8 | 86 | 10 | 17 | 16 |
| senkyunolide A | C12H16O2 | 7.51 | 193.027 | 91.0 | 86 | 10 | 27 | 10 |
| butylphthalide* | C12H14O2 | 7.87 | 191.058 | 173.0 | 56 | 10 | 13 | 18 |
| butylphthalide | C12H14O2 | 7.87 | 191.058 | 144.9 | 56 | 10 | 21 | 16 |
| butylidenephthalide* | C12H12O2 | 9.99 | 189.021 | 171.0 | 86 | 10 | 19 | 20 |
| butylidenephthalide | C12H12O2 | 9.99 | 189.021 | 127.9 | 86 | 10 | 33 | 14 |
| senkyunolide I | C12H16O4 | 4.01 | 225.133 | 207.0 | 91 | 10 | 9 | 24 |
| senkyunolide I* | C12H16O4 | 4.01 | 225.133 | 165.0 | 91 | 10 | 23 | 18 |
| senkyunolide H | C12H16O4 | 4.19 | 225.121 | 207.0 | 91 | 10 | 9 | 24 |
| senkyunolide H* | C12H16O4 | 4.19 | 225.121 | 189.1 | 91 | 10 | 25 | 20 |
| ligustilide | C12H14O2 | 9.72 | 191.049 | 115.0 | 126 | 10 | 47 | 50 |
| ligustilide* | C12H14O2 | 9.72 | 191.049 | 91.0 | 126 | 10 | 31 | 40 |
| Clarithromycin | C38H69NO13 | 5.63 | 748.364 | 590.0 | 31 | 10 | 31 | 26 |

* The quantification ion

**Table S4** UPLC-ESI-MS/MS method validation parameters for the tested components

| Analytes | Regression  Equation | R^2^ | Linear  Range  (µg/mL) | LOD  (ng/mL) | LOQ  (ng/mL) | Precision | | Stability (RSD, %) | Reproducibility (RSD, %) | Recovery (n = 6) | |
| --- | --- | --- | --- | --- | --- | --- | --- | --- | --- | --- | --- |
|  |  |  |  |  |  | Intraday  (n = 6) | Interday  (n = 18) |  |  | Mean,% | RSD,% |
| ligustilide | y=0.0007x+0.0076 | 0.9959 | 0.0068-778.5 | 6.1902 | 20.6339 | 0.48 | 0.87 | 1.10 | 1.92 | 99.31 | 1.77 |
| senkyunolide H | y=0.0094x+0.0225 | 0.9920 | 0.0051-20.64 | 7.2548 | 24.1828 | 0.79 | 1.07 | 1.41 | 2.76 | 98.23 | 2.09 |
| senkyunolide I | y=0.0056x-0.0697 | 0.9970 | 0.0049-20.06 | 18.8682 | 62.8941 | 1.37 | 1.44 | 0.69 | 1.44 | 99.17 | 1.14 |
| butylidenephthalide | y=0.0052x+0.064 | 0.9935 | 0.0047-19.41 | 5.1192 | 17.0640 | 1.95 | 1.96 | 1.56 | 1.95 | 101.32 | 1.65 |
| butylphthalide | y=0.0099x+0.1296 | 0.9978 | 0.0086-17.52 | 3.1902 | 10.6341 | 1.26 | 1.66 | 0.82 | 1.66 | 100.44 | 1.19 |
| senyunolide A | y=0.0213x+0.517 | 0.9940 | 0.0077-15.75 | 0.1846 | 0.6152 | 1.31 | 1.69 | 1.54 | 1.85 | 98.61 | 1.47 |

**Table S5** The correlation between bacterial on genus level and phthalides accumulation

|  | ligustilide | butylidenephthalide | senkyunolide_H | senkyunolide_I | butylphthalide | senyunolide_A |
| --- | --- | --- | --- | --- | --- | --- |
| g__Rhizobium | -0.78269 | -0.57977 | 0.115954 | 0.115954 | -0.63775 | -0.66674 |
| g__Pseudomonas | -0.77143 | -0.25714 | 0.257143 | 0.428571 | -0.48571 | -0.42857 |
| g__Flavobacterium | -0.42857 | -0.48571 | -0.31429 | -0.14286 | -0.25714 | -0.65714 |
| g__Gaiella | -0.37143 | -0.54286 | -0.37143 | -0.31429 | 0.6 | 0.314286 |
| g__Sphingomonas | -0.37143 | -0.82857 | -0.48571 | -0.65714 | 0.428571 | -0.2 |
| g__Streptomyces | -0.31429 | -0.71429 | -0.54286 | -0.37143 | -0.25714 | -0.31429 |
| g__Nitrospira | -0.08571 | -0.37143 | -0.54286 | -0.37143 | 0.771429 | 0.485714 |
| g__Bacillus | -0.02857 | -0.08571 | 0.085714 | -0.08571 | 0.371429 | 0.542857 |
| g__Bradyrhizobium | 0.028571 | 0.771429 | 0.6 | 0.771429 | 0.314286 | 0.828571 |
| g__Microlunatus | 0.085714 | 0.314286 | -0.02857 | 0.257143 | 0.657143 | 0.942857 |
| g__Pseudonocardia | 0.085714 | 0.714286 | 0.542857 | 0.6 | 0.028571 | 0.085714 |
| g__RB41 | 0.257143 | -0.2 | -0.54286 | -0.6 | 0.828571 | 0.085714 |
| g__H16 | 0.257143 | -0.02857 | -0.37143 | -0.08571 | -0.02857 | 0.428571 |
| g__Mycobacterium | 0.428571 | 0.428571 | 0.085714 | 0.142857 | 0.314286 | 0.828571 |
| g__Roseiflexus | 0.485714 | 0.657143 | 0.142857 | 0.314286 | 0.428571 | 0.942857 |
| g__Candidatus_Alysiosphaera | 0.542857 | 0.942857 | 0.428571 | 0.485714 | 0.257143 | 0.542857 |
| g__Candidatus_Xiphinematobacter | 0.6 | 0.257143 | -0.08571 | -0.37143 | 0.371429 | -0.02857 |
| g__Solirubrobacter | 0.771429 | 0.257143 | -0.08571 | -0.37143 | -0.2 | -0.2 |
| g__Nocardioides | 0.885714 | 0.371429 | -0.14286 | -0.31429 | -0.08571 | 0.085714 |
| g__Blastococcus | 0.942857 | 0.6 | -0.08571 | -0.14286 | 0.028571 | 0.2 |

Supplemental Experimental Procedures

*Sample collection*

All root and soil samples were consistent with plant samples collected from Min County, Gansu Province and Diqing, Yunnan Province. Min County belongs to the temperate semi-humid to alpine and humid climate transition zone, with high and cold dampness and good vegetation coverage. The annual average temperature is 5.5°C, the annual precipitation is 635 mm, the average relative humidity is 68%, and the average annual sunshine is 2214.9 h. Diqing City is located in the low latitude plateau and belongs to the subtropical and temperate monsoon plateau mountain climate of the Huaxi-like Kangdian District of Tibet. The annual average temperature is 6.9°C, the annual average precipitation is 938.11 mm, the average relative humidity is 70%，and the average annual sunshine is 2104.5 h.

*Standard preparation*

Reference standards, including ligustilide (1), butylphthalide (2), butylidenephalide (3), senkyunolide H (4), senkyunolide I (5), and senkyunolide A (6), which each had a purity > 98%, were obtained from Liangwei Biochemical Reagent, Ltd. (Nanjing, China). The chemical structure of each reference standard is provided (Figure S1).

Standard solutions of ligustilide, butylphthalide, butylidenephthalide, senkyunolide H, senkyunolide I, senkyunolide A, and clarithromycin (IS) were prepared in methanol. The concentrations were as follows: 432.5 μg/mL ligustilide, 545 μg/mL butylphthalide, 277 μg/mL butylidenephalide, 321 μg/mL senkyunolide H, 312 μg/mL senkyunolide I, 490 μg/mL senkyunolide A, and 100 ng/mL clarithromycin (IS).

*UHPLC chromatographic condition*

Chromatographic analyses were performed using a Waters Acquity UPLC system (Waters, Corp., MA, USA), which consisted of a binary pump solvent management system, autosampler, and online degasser. An Acquity BEH C_18_ column (100 mm × 2.1 mm × 1.7 μm) was used for all processes. The mobile phase comprised A (0.1% formic acid, v/v) and B (acetonitrile) with a gradient elution as follows: 0–5 min, 95–43% B; 5–9 min, 43% B; 9–12 min, 43–70% B; 12–14 min, 70–95% B; 14–15 min, 95–5% B; and 16 min, 95% B. The flow rate was set at 0.4 mL/min. The column temperature was conditioned at 35°C, and the injection volume was 2 µL.

*UHPLC-QqQ-MS chromatographic condition*

Mass spectrometry (MS) was carried out using an AB SCIEX Triple Quad 6500 plus (AB SCIEX Corp., MA, USA) with electrospray ionization (ESI). The ESI-MS spectra were acquired in the positive ion multiple reaction monitoring (MRM) mode. The MS parameters were optimized and set as follows: capillary voltage, 5 kV; desolvation gas flow rate, 1000 L/h at 550°C; and cone gas flow, 50 L/h at 150°C. The declustering potential (DP) and collision energy (CE) were set to match the MRM of each marker (Weng et al., 2018). The dwell time was automatically set by the MultiQuant software. The raw data were processed with MultiQuant v3.0.2 (AB SCIEX Corp., MA, USA) and SPSS v21.0 software (SPSS Inc., Chicago, IL).

*Methodology validation*

The calibration curve was constructed by plotting the peak area against the corresponding concentration. The standard curves of six standards were built and the regression equations were calculated. The precision of the method was evaluated by the standard solution. The solution was subjected to three successive days of six successive injections on the same day. The stability was assessed by analyzing sample solutions of A. sinensis (M-ZC-G-3) at 0, 4, 8, 12, 16, 20, and 24 h. The reproducibility comprised six independent samples that were prepared for repeated detection according to the above methods. The recovery test was evaluated by adding three standard solutions of known concentrations (80%, 100%, and 120%) of mixed standard solutions into 0.5 g of *A. sinensis* (M-ZC2-G-3). All the above experiments were conducted in triplicate and expressed as the relative standard deviation (RSD).

*Illumina MiSeq sequencing and data analysis*

The V3-V4 hypervariable regions of the bacteria 16S rDNA were amplified with the primers, 338F (5’-ACTCCTACGG GAGGCAGCAG-3’) and 806R (5’-GGACTACHVGGGT WTCTAAT-3’), by using a Gene Amp 9700 thermocycler PCR system (Applied Biosystems, USA) (Chen et al., 2018a). PCR reactions were conducted using the following procedure: 3 min of denaturation at 95°C, 27 cycles for 30 s at 95°C, 30 s of annealing at 55°C, 45 s of elongation at 72°C, and a final extension at 72°C for 10 min (Chen et al., 2018b). Paired-end Illumina reads were subjected to trimming, filtering, and quality control using the cloud platform (https://www.i-sanger.com/) provided by Shanghai Majorbio Bio-Pharm Technology Co., Ltd.

OTUs were used for alpha diversity and beta diversity. The Sob, Shannon, Simpson, ACE, Chao and sequencing depths (Good’s coverage) were showed in the alpha diversity. The principal coordinates analysis (PCoA), nonmetric multidimensional scaling (NMDS) diagrams and ANOSIM were showed in the beta diversity. They were generated using the R v3.5.1 package.

*Bacterial strains culture*

Two stains bacterial, XG-2 and XG-3, isolated from the *A. sinensis* soils of Min Country were identified as the *Bacillus subtilis* and *Bacillus velezensis*, based on 16S rDNA sequence. The *Bacillus* were grown on 250 mL Erlenmeyer baffle flasks containing 50 mL of the Luria-Bertani medium with a rotary shaker at 160 rpm and 30°C for 24h. Then some cultures ( XG-2-J/XG-3-J ) were centrifuged at 5000 g for 20 min, the cell pellets were suspended in sterile distilled water and adjusted to an OD600 of 0.5, which corresponded to a 10^8^ colony-forming unit per milliliter. On the other hand, some cultures ( XG-2-JY/XG-3-JY ) were adjusted to an OD600 of 0.5 by sterile distilled water.

*The tissue culture seedlings of A. sinensis growth*

Seeds of *A. sinensis* which were collected from Min Country, Dingxi city, Gansu Province, China, were used in experiments. Seeds were surface sterilized by immersing samples in 75% (v/v) ethanol for 1 min and 0.1% HgCl_2_ (w/v) for 11 min, then subsequently rinsed five times in distilled H_2_O. Seeds were then germinated on basal Murashige and Skoog (MS) medium supplemented with 3% (w/v) sucrose and 0.77% (w/v) agar power as solidification agents. Then medium were adjustment of pH (5.6-5.8) and sterilization (autoclaving at 120°C for 20 min). Each flask of 60 mL medium was inoculated with five seeds and germinated seeds were allowed to grow in culture room at 23 ± 1°C under a 14-hour photoperiod and 2000 lx light intensity. (Benzle K, et al., 2017).

Reference

Benzle K, Cornish K. Improved axenic hydroponic whole plant propagation for rapid production of roots as transformation target tissue. *Plant Methods*, 2017, 13(1):37. doi: 10.1186/s13007-017-0189-z.

Chen, Y., Chang, S.K.C., Chen, J., Zhang, Q., and Yu, H. (2018a). Characterization of microbial community succession during vermicomposting of medicinal herbal residues. *Bioresour Technol* 249, 542-549. doi: 10.1016/j.biortech.2017.10.021.

Chen, Y.J., Wu, H., Wu, S.D., Lu, N., Wang, Y.T., et al. (2018b). Parasutterella, in association with irritable bowel syndrome and intestinal chronic inflammation. *J Gastroenterol Hepatol* 33, 1844-1852. doi: 10.1111/jgh.14281.

Weng, Z., Zeng, F., Zhu, Z., Qian, D., Guo, S., et al. (2018). Comparative analysis of sixteen flavonoids from different parts of Sophora flavescens Ait. by ultra high-performance liquid chromatography-tandem mass spectrometry. *J Pharm Biomed Anal* 156, 214-220. doi: 10.1016/j.jpba.2018.04.046.
